# Developing and validating a machine learning model to predict multidrug-resistant *Klebsiella pneumoniae*-related septic shock

**DOI:** 10.3389/fimmu.2024.1539465

**Published:** 2025-01-10

**Authors:** Shengnan Pan, Ting Shi, Jinling Ji, Kai Wang, Kun Jiang, Yabin Yu, Chang Li

**Affiliations:** ^1^ Department of Medical Laboratory, The Affiliated Huai’an No. 1 People’s Hospital of Nanjing Medical University, Huai’an, Jiangsu, China; ^2^ Department of Hepatobiliary and Pancreatic Surgery, The Affiliated Huai’an No. 1 People’s Hospital of Nanjing Medical University, Huai’an, Jiangsu, China; ^3^ Department of Rheumatology, The Affiliated Huai’an No. 1 People’s Hospital of Nanjing Medical University, Huai’an, Jiangsu, China

**Keywords:** multidrug-resistant *Klebsiella pneumoniae*, septic shock, machine learning, predictive model, risk factors

## Abstract

**Background:**

Multidrug-resistant Klebsiella pneumoniae (MDR-KP) infections pose a significant global healthcare challenge, particularly due to the high mortality risk associated with septic shock. This study aimed to develop and validate a machine learning-based model to predict the risk of MDR-KP-associated septic shock, enabling early risk stratification and targeted interventions.

**Methods:**

A retrospective analysis was conducted on 1,385 patients with MDR-KP infections admitted between January 2019 and June 2024. The cohort was randomly divided into a training set (n = 969) and a validation set (n = 416). Feature selection was performed using LASSO regression and the Boruta algorithm. Seven machine learning algorithms were evaluated, with logistic regression chosen for its optimal balance between performance and robustness against overfitting.

**Results:**

The overall incidence of MDR-KP-associated septic shock was 16.32% (226/1,385). The predictive model identified seven key risk factors: procalcitonin (PCT), sepsis, acute kidney injury, intra-abdominal infection, use of vasoactive medications, ventilator weaning failure, and mechanical ventilation. The logistic regression model demonstrated excellent predictive performance, with an area under the receiver operating characteristic curve (AUC) of 0.906 in the training set and 0.865 in the validation set. Calibration was robust, with Hosmer-Lemeshow test results of *P* = 0.065 (training) and *P* = 0.069 (validation). Decision curve analysis indicated substantial clinical net benefit.

**Conclusion:**

This study presents a validated, high-performing predictive model for MDR-KP-associated septic shock, offering a valuable tool for early clinical decision-making. Prospective, multi-center studies are recommended to further evaluate its clinical applicability and effectiveness in diverse settings.

## 1 Introduction

Multidrug-resistant Klebsiella pneumoniae (MDR-KP) infections have become a critical global health threat, escalating in both incidence and mortality, and imposing substantial burdens on healthcare systems worldwide (1). In particular, healthcare infrastructures in resource-limited settings face increasing strain due to the complexity and severity of MDR-KP infections (2). Prolonged hospitalizations, the need for advanced diagnostics, and the administration of high-cost, broad-spectrum antibiotics can significantly increase direct healthcare expenditures. These pressures not only intensify the economic burden on healthcare institutions but also limit the optimal allocation of scarce resources, ultimately hindering patient care and system resilience (3). The surge in MDR-KP cases is primarily attributed to widespread antimicrobial use, which presents significant therapeutic dilemmas in contemporary clinical practice (4). Notably, MDR-KP infections are associated with mortality rates ranging from 40% to 50%, with those involving bloodstream infections experiencing even higher mortality rates up to 70% (5). Septic shock represents one of the most severe complications of MDR-KP infection, often leading to multiple organ dysfunction and increased mortality (6).

The high morbidity and mortality of MDR-KP infections directly correlates with their complex antimicrobial resistance mechanisms. MDR-KP demonstrates adaptability primarily through the production of β-lactamase enzymes, including ESBLs and carbapenemases, leading to broad-spectrum resistance (7). Additional resistance mechanisms include altered outer membrane protein expression and enhanced efflux pump activity (8). These combined resistance mechanisms often render conventional antibiotics ineffective, compromising infection control and increasing the risk of septic shock.

Current MDR-KP research focuses on three main areas: epidemiology, resistance mechanisms, and treatment optimization. Key risk factors for MDR-KP infections include prolonged hospitalization, invasive procedures, and immunocompromised status (9). Treatment research has explored various strategies, including antimicrobial combinations, new antibiotic development, and alternative approaches such as bacteriophage therapy (10). However, there remains a significant gap in developing predictive models for MDR-KP-associated septic shock risk. Early identification and intervention in high-risk cases significantly improve patient outcomes (11). This highlights the need for accurate predictive models to guide clinical decisions and improve prognosis. Although general sepsis prediction tools like qSOFA provide a quick bedside assessment for sepsis and related complications (12), these generic tools may not adequately capture the unique pathophysiology of MDR-KP infections, limiting their accuracy in predicting MDR-KP-specific septic shock risk.

Machine learning has increasingly been applied in medicine for disease risk prediction and prognosis (13–16). These methods excel at analyzing complex clinical data, identifying hidden risk factors and patterns while improving predictive accuracy (17). In infectious diseases, machine learning has shown success in sepsis prediction (18) and antimicrobial resistance forecasting (19). In contrast to traditional statistical approaches, machine learning methods are particularly suitable for this study due to their ability to handle high-dimensional, complex clinical data and uncover intricate nonlinear relationships among variables without stringent parametric assumptions. While traditional statistical models (e.g., logistic regression, Cox proportional hazards models) typically depend on predefined assumptions about data distributions and variable interactions, machine learning approaches autonomously identify and weigh critical predictors, effectively manage collinearity and nonlinearities, and adapt to evolving data patterns. This flexibility and robustness can substantially enhance predictive accuracy and stability, enabling a more comprehensive understanding of multifactorial conditions such as MDR-KP-associated septic shock. However, current MDR-KP prediction models primarily rely on conventional statistics, underutilizing the potential of Big Data analytics and Artificial Intelligence (AI). Current predictive modeling research has several important methodological limitations. First, studies often use limited sample populations, which may affect model stability and generalizability. Second, many models incorporate an insufficient range of predictive variables, potentially missing key risk factors. Additionally, model validation often lacks comprehensive calibration metrics and robust clinical utility assessment. These limitations may reduce the models’ practical utility in real clinical settings.

In this study, we aim to develop a machine learning-based predictive model for MDR-KP-associated septic shock risk, integrating clinical and laboratory parameters. The model incorporates two key improvements: large-scale sampling for model stability and comprehensive risk factors, including demographics, comorbidities, laboratory data, and treatment variables. Model validation employs multiple metrics, including discrimination, calibration, and clinical decision curve analysis, to assess both predictive accuracy and clinical utility.

## 2 Materials and methods

### 2.1 Study data

This investigation received ethical authorization from the Ethics Committee of the Affiliated Huai’an No. 1 People’s Hospital of Nanjing Medical University (approval number: KY-2024-355-01). Informed consent was waived due to the retrospective design. Data anonymization and confidentiality were maintained according to the Declaration of Helsinki.

We conducted a retrospective analysis of clinical data from 1,475 patients infected with MDR-KP who were admitted to The Affiliated Huai’an No.1 People’s Hospital of Nanjing Medical University from January 2019 to June 2024. A total of 1,385 patients met the stringent inclusion criteria and were subsequently analyzed statistically. The criteria for patient inclusion required individuals to be adults aged 18 or older, to have a confirmed MDR-KP infection, and to possess comprehensive clinical documentation, including demographic data (such as gender and age) and laboratory parameters. Exclusion criteria were defined to omit patients with psychiatric or cognitive disorders, those hospitalized for less than 24 hours, patients with concurrent malignancies, cases where MDR-KP was isolated outside the designated timeframe (prior to admission, within 48 hours following admission, or more than 72 hours after discharge), or individuals suffering from non-MDR-KP-induced septic shock.

### 2.2 Study design

We developed a machine learning-based predictive model to determine the risk of Septic Shock in patients with Multidrug-resistant Klebsiella pneumoniae (MDR-KP). This retrospective study analyzed data from 1,385 confirmed cases of MDR-KP infections, defining MDR-KP as strains of K. pneumoniae resistant to at least one agent in three or more antimicrobial categories (20). Septic shock was characterized following the Sepsis-3 criteria (21). Clinical data, extracted from electronic health records, encompassed a wide range of variables, including demographics, laboratory indices, treatments, comorbidities, infection sites, metrics of organ dysfunction, and complications. The dataset was randomly split into training (70%) and test (30%) sets.

### 2.3 Machine learning

**Figure 1:**
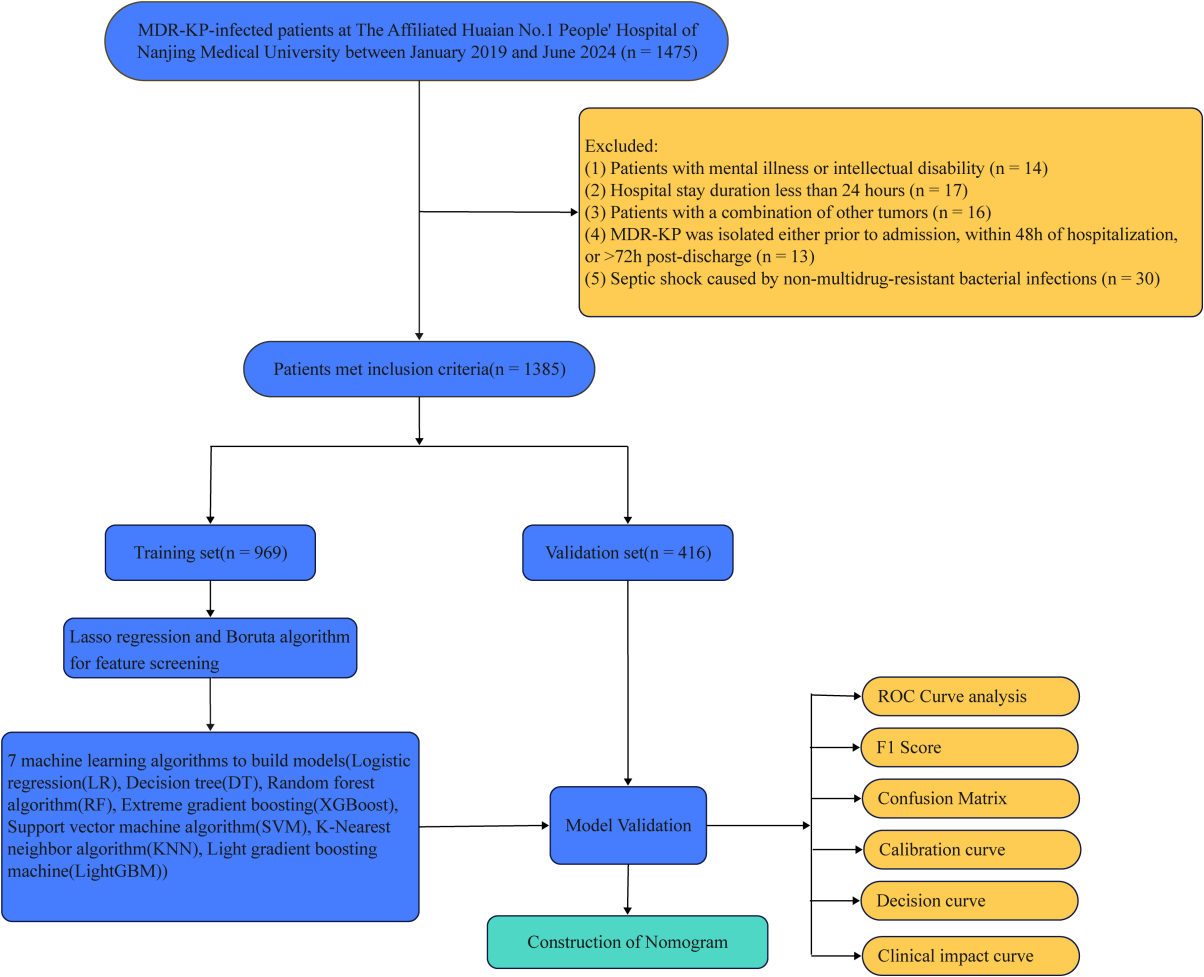
Research flowchart.

Feature selection combined Boruta algorithm and LASSO regression (22–24). Multiple machine learning models were evaluated: Logistic Regression (LR), Decision Trees (DT), Random Forests (RF), eXtreme Gradient Boosting (XGBoost), Support Vector Machines (SVM), K-Nearest Neighbors (KNN), and Light Gradient Boosting Machine (LightGBM), using 10-fold cross-validation (25–27). Model performance was assessed through area under the curve (AUC), accuracy, sensitivity, specificity, predictive values, F1 score, calibration curves, and clinical impact analysis. A nomogram was developed to visualize feature importance and prediction mechanisms. The workflow is illustrated in **Figure 1**.

### 2.4 Research variables

A total of 99 clinical predictors across seven major categories were analyzed. These included demographic factors (gender, age); laboratory parameters (SO_2_, pH, HCO_3_^-^, BE, PO_2_, PCO_2_, AG, WBC count, HGB, RBC count, HCT, RDW-SD, RDW-CV, MCV, MCHC, MCH, PLT count, PDW, MPV, neutrophils [percentage and count], monocytes [percentage and count], lymphocytes [percentage and count], hs-CRP, PCT, Urea, creatinine, UA, CYSC, Serum CO_2_, TBIL, DBIL, AST, ALT, ALP, γ-GGT, TP, ALB, AFU, ADA, CHE, PA, HBDH, TG, CHOL, CK, LDH, Na, Ca, P, K, Mg, TT, PT, Fibrinogen, D-dimer, NT-proBNP); treatment interventions (surgery, endotracheal intubation, mechanical ventilation, ventilator weaning failure, vasoactive medications, CRRT use, ECMO use, anticoagulant use, tracheostomy); comorbidities (hypertension, diabetes mellitus, diabetes-related complications, hyperlipidemia, hyperlactatemia, coronary heart disease, atrial fibrillation, chronic renal insufficiency, heart failure, anemia); infection sites (liver abscess, biliary tract infection, intra-abdominal infection, pneumonia, urinary tract infection, intracranial infection); acute organ injury (acute liver injury, acute kidney injury, ARDS, MODS, altered mental status, stroke, metabolic encephalopathy, hepatic encephalopathy, ventricular fibrillation, cardiac arrest); and complications (coagulopathy, DIC, sepsis). Demographic and comorbidity data were collected before MDR-KP infection onset. Laboratory parameters were based on initial post-admission results.

### 2.5 Diagnostic criteria of septic shock

Septic shock diagnosis followed the Third International Consensus Definitions for Sepsis and Septic Shock (Sepsis-3) criteria (21). Diagnostic requirements included: 1) suspected or documented infection; 2) persistent hypotension requiring vasopressors to maintain mean arterial pressure ≥65 mmHg; and 3) serum lactate level >2 mmol/L despite adequate volume resuscitation. Alternative causes of hypotension were excluded. While noted, additional features such as altered mental status, tachypnea, decreased urine output, and poor peripheral circulation were not mandatory for diagnosis.

### 2.6 Feature selection techniques

To refine our predictive model, we utilized the Least Absolute Shrinkage and Selection Operator (LASSO) regression (28), leveraging the ‘glmnet’ package in R with settings adjusted for binomial distribution (α = 1) (29, 30). This method determined optimal lambda values via cross-validation—lambda.min for the minimal error and lambda.1se to ensure parsimony—thereby retaining variables with non-zero coefficients which helped mitigate multicollinearity and overfitting in our high-dimensional dataset (31, 32). Additionally, we implemented the Boruta algorithm, a Random Forest-based feature selection method (33, 34). This algorithm assesses feature importance by creating ‘shadow attributes’ for each original feature, iteratively comparing their importance through 500 iterations or until stability is achieved (35, 36). Important features were identified as those consistently outperforming their shadow counterparts, with results processed using the ‘attStats’ and custom ‘adjustdata’ functions to ensure robust selection and minimize false negatives (37, 38).

### 2.7 Machine learning algorithms

Several machine learning algorithms were used to predict MDR-KP-associated septic shock risk. Logistic Regression (LR) was applied with L2 regularization to model the binary outcomes, optimizing hyperparameters like regularization factor and iteration limits for balance and precision (39–41). The Decision Tree model utilized the CART algorithm, setting parameters to maintain a balance between complexity and interpretability (37, 42, 43). The Random Forest algorithm was configured with parameters to capture non-linear associations and enhance generalizability (44, 45). For robust pattern recognition, the Extreme Gradient Boosting (XGBoost) model was optimized with specific parameters for tree depth, learning rate, and regularization (46, 47). The Support Vector Machine (SVM) algorithm employed a Radial Basis Function (RBF) kernel, focusing on optimizing the decision boundary in high-dimensional space (24, 48, 49). K-Nearest Neighbors (KNN) used a simple majority voting mechanism with uniform weighting, and the Light Gradient Boosting Machine (LightGBM) was optimized for computational efficiency and effective pattern recognition, making extensive use of gradient boosting technology (50–52). Each model’s configuration aimed to effectively identify risk factors and predict outcomes accurately within our dataset.

### 2.8 Decision curve analysis

In this study, we employed decision curve analysis (DCA) to evaluate the clinical utility of our predictive model (22, 53). The fundamental principle of DCA is to compare the net benefit derived from “intervening based on the model’s predictions” with that of two extreme strategies “treating all patients” or “treating none” across a range of clinically relevant threshold probabilities. By doing so, DCA provides insights into the model’s incremental value in real-world decision-making scenarios. More specifically, DCA integrates the predicted probability of a patient developing MDR-KP-associated septic shock with the clinician’s predetermined threshold probability for initiating intervention (e.g., 10% or 20%). For instance, if a physician chooses to escalate care, such as intensifying monitoring, adjusting antimicrobial regimens, or providing early hemodynamic support only when a patient’s risk surpasses a certain threshold, DCA graphically illustrates whether using the model at that threshold yields additional clinical benefit compared to not using it.

### 2.9 Statistical analysis

Statistical analyses were performed using SPSS 25.0 (IBM Corp., NY, USA) and R 4.3.2 (R Foundation, Austria). Normally distributed continuous variables were expressed as mean ± standard deviation and compared using independent t-tests, while non-normal variables were presented as median (interquartile range) with Mann-Whitney U tests. Categorical variables were analyzed using chi-square or Fisher’s exact tests as appropriate. Statistical significance was set at P < 0.05 (54, 55).

## 3 Results

### 3.1 Characteristics of patients with MDR-KP infections in a retrospective study

Among the 1,475 patients with MDR-KP infections admitted to the First Affiliated Hospital of Nanjing Medical University Huai’an from January 2019 and June 2024, 1,385 patients were eventually eligible and included in the statistical analysis (**Figure 1**). A total of 1,385 patients with MDR-KP infections were divided into a training set consisting of 969 patients and a validation set consisting of 416 patients, following a ratio of 7:3. Statistical analysis revealed no significant differences between the two groups.

### 3.2 Incidence of MDR-KP-related septic shock

The total incidence of MDR-KP-related septic shock was 16.32% (226/1,385), with comparable rates in the training [16.82% (163/969)] and validation [15.14% (63/416)] sets. Significant differences (*P*<0.05) between groups in the training set were observed across multiple parameters (**Table 1**). Laboratory indices showed differences in HCO3-, BE, WBC, RDW (SD and CV), blood cell counts (PLT, neutrophils, monocytes, lymphocytes), monocytes percentage, PCT, renal markers (urea, creatinine, UA, CYSC), liver function tests (TBIL, DBIL, AST, TP, ALB, AFU, CHE), metabolic parameters (PA, α-HBDH, CHOL, LDH), heart failure indicators (NT-proBNP), electrolytes (Ca^2+^, K^+^), and coagulation markers (PT, D-dimer). Clinical parameters including age, along with interventions such as surgery, endotracheal intubation, mechanical ventilation, ventilator weaning status, vasoactive medications, CRRT, and anticoagulation also differed significantly. Additionally, notable differences were found in comorbidities and complications, encompassing diabetes-related complications, hyperlipidemia, hyperlactatemia, intra-abdominal infection, pneumonia, acute liver/kidney injury, ARDS, MODS, stroke, cardiac arrest, coagulopathy, DIC, and sepsis.

**Table 1:** Baseline characterization and comparison.

Variables	Total (N = 969)	MDR-KP-related Septic Shock		*P* value
		No (N = 806)	Yes (N = 163)	
Demography				
Age (year)	65.00 (54.00, 75.00)	64.00 (54.00, 74.00)	68 (56.00, 76.50)	0.036
Gender, *n* (%)				0.645
Female	285 (29.41)	240 (29.78)	45 (27.61)	
Male	684 (70.59)	566 (70.22)	118 (72.39)	
Laboratory results				
HCO_3_^-^(mmol/L)	24.80 (21.00, 28.90)	25.15 (21.33, 29.17)	22.80 (20.40, 26.50)	0.004
BE (mmol/L)	0.60 (-3.90, 4.40)	1.05 (-3.77, 5.07)	‘-2.10 (-4.50, 1.90)	0.003
WBC count (10^9^/L)	10.38 (7.12, 14.68)	10.59 (7.34, 14.76)	9.07 (6.30, 13.31)	0.010
RDW-SD (fL)	44.60 (41.90, 48.90)	44.50 (41.70, 48.60)	45.40 (43.05, 49.60)	0.016
RDW-CV (%)	13.50 (12.80, 14.60)	13.50 (12.80, 14.60)	13.80 (13.10, 15.05)	0.003
PLT count(10^9^/L)	191.00 (132.00, 249.00)	197.50 (141.25, 255.75)	140.00 (93.50, 218.00)	< 0.001
Neutrophils count (10^9^/L)	8.51 (5.37, 12.67)	8.74 (5.48, 12.85)	7.78 (4.48, 11.84)	0.027
Monocytes percentage (%)	5.30 (3.60, 7.30)	5.50 (3.80, 7.50)	4.30 (2.60, 6.55)	< 0.001
Monocytes count (10^9^/L)	0.53 (0.35, 0.78)	0.55 (0.38, 0.80)	0.41 (0.22, 0.66)	< 0.001
Lymphocytes count (10^9^/L)	0.91 (0.59, 1.36)	0.93 (0.62, 1.39)	0.79 (0.41, 1.16)	< 0.001
PCT	0.40 (0.09, 1.66)	0.39 (0.08, 1.06)	1.67 (0.36, 14.59)	< 0.001
Urea (mmol/L)	6.78 (5.01, 10.07)	6.62 (4.85, 9.29)	9.22 (6.26, 13.98)	< 0.001
Creatinine (μmol/L)	70.40 (52.60, 94.00)	70.40 (51.00, 90.45)	79.80 (63.10, 136.05)	< 0.001
UA (μmol/L)	241.00 (165.00, 341.00)	241.00 (162.00, 332.75)	272.00 (182.50, 379.00)	< 0.001
CYSC (mg/L)	0.95 (0.75, 1.26)	0.95 (0.73, 1.21)	1.14 (0.88, 1.61)	< 0.001
TBIL (μmol/L)	11.90 (8.00, 18.20)	11.90 (7.62, 17.40)	15.70 (9.65, 22.75)	< 0.001
DBIL(μmol/L)	5.60 (3.90, 8.40)	5.50 (3.80, 7.80)	7.70 (5.20, 13.05)	< 0.001
AST (U/L)	27.85 (18.80, 49.40)	27.85 (18.40, 46.92)	32.20 (20.85, 76.00)	0.006
TP (g/L)	59.32 ± 9.04	60.01 ± 8.70	55.87 ± 9.87	< 0.001
ALB (g/L)	34.57 ± 6.66	34.92 ± 6.46	32.86 ± 7.34	0.001
AFU (U/L)	18.70 (15.10, 23.70)	18.70 (15.00, 22.98)	20.10 (16.50, 27.25)	< 0.001
CHE (U/L)	4997.00 (3581.00, 6651.00)	4998.50 (3802.25, 6737.25)	4210.00 (2841.00, 5754.00)	< 0.001
PA (mg/dL)	157.85 (107.20, 201.20)	158.80 (114.80, 204.07)	120.50 (82.75, 190.65)	< 0.001
α-HBDH (U/L)	188.00 (149.00, 257.00)	188.00 (147.00, 242.00)	207.00 (163.00, 297.50)	< 0.001
CHOL (mmol/L)	3.28 (2.58, 4.09)	3.28 (2.74, 4.13)	2.81 (2.04, 3.74)	< 0.001
LDH (U/L)	241.00 (195.00, 326.00)	241.00 (193.00, 318.75)	262.00 (210.00, 383.00)	< 0.001
Ca (mmol/L)	2.13 (2.02, 2.24)	2.13 (2.03, 2.26)	2.09 (1.94, 2.20)	< 0.001
K (mmol/L)	4.01 (3.7, 4.31)	4.00 (3.67, 4.26)	4.14 (3.78, 4.56)	< 0.001
PT (s)	14.00 (12.90, 15.30)	14.00 (12.70, 15.00)	15.10 (13.50, 17.60)	< 0.001
D-dimer (mg/L)	2.79 (1.46, 6.63)	2.79 (1.38, 5.82)	3.31 (1.77, 8.52)	0.006
NT-proBNP (pg/mL)	863.55 (471.00, 1528.00)	863.55 (435.47, 1185.00)	1264.00 (742.55, 4236.00)	< 0.001
Treatment, *n* (%)				
Surgery, *n* (%)				0.041
No	594 (61.30)	482 (59.80)	112 (68.71)	
Yes	375 (38.70)	324 (40.20)	51 (31.29)	
Endotracheal intubation, *n* (%)				< 0.001
No	640 (66.05)	576 (71.46)	64 (39.26)	
Yes	329 (33.95)	230 (28.54)	99 (60.74)	
Mechanical ventilation, *n* (%)				< 0.001
No	515 (53.15)	481 (59.68)	34 (20.86)	
Yes	454 (46.85)	325 (40.32)	129 (79.14)	
Ventilator weaning failure, *n* (%)				< 0.001
No	924 (95.36)	794 (98.51)	130 (79.75)	
Yes	45 (4.64)	12 (1.49)	33 (20.25)	
Vasoactive medications, *n* (%)				< 0.001
No	830 (85.66)	739 (91.69)	91 (55.83)	
Yes	139 (14.34)	67 (8.31)	72 (44.17)	
CRRT use, *n* (%)				< 0.001
No	891 (91.95)	766 (95.04)	125 (76.69)	
Yes	78 (8.05)	40 (4.96)	38 (23.31)	
ECMO use, *n* (%)				0.071
No	959 (98.97)	800 (99.26)	159 (97.55)	
Yes	10 (1.03)	6 (0.74)	4 (2.45)	
Anticoagulant use, *n* (%)				0.031
No	776 (80.08)	656 (81.39)	120 (73.62)	
Yes	193 (19.92)	150 (18.61)	43 (26.38)	
Diabetes-related complications, *n* (%)				< 0.001
No	931 (96.08)	784 (97.27)	147 (90.18)	
Yes	38 (3.92)	22 (2.73)	16 (9.82)	
Hyperlipidemia, *n* (%)				0.005
No	958 (98.86)	801 (99.38)	157 (96.32)	
Yes	11 (1.14)	5 (0.62)	6 (3.68)	
Hyperlactatemia, *n* (%)				0.010
No	953 (98.35)	797 (98.88)	156 (95.71)	
Yes	16 (1.65)	9 (1.12)	7 (4.29)	
Intra-abdominal infection, *n* (%)				< 0.001
No	939 (96.90)	796 (98.76)	143 (87.73)	
Yes	30 (3.10)	10 (1.24)	20 (12.27)	
Pneumonia, *n* (%)				0.049
No	253 (26.11)	221 (27.42)	32 (19.63)	
Yes	716 (73.89)	585 (72.58)	131 (80.37)	
Acute organ injury, *n* (%)				
Acute liver injury, *n* (%)				< 0.001
No	823 (84.93)	709 (87.97)	114 (69.94)	
Yes	146 (15.07)	97 (12.03)	49 (30.06)	
Acute kidney injury, *n* (%)				< 0.001
No	775 (79.98)	695 (86.23)	80 (49.08)	
Yes	194 (20.02)	111 (13.77)	83 (50.92)	
ARDS, *n* (%)				< 0.001
No	576 (59.44)	520 (64.52)	56 (34.36)	
Yes	393 (40.56)	286 (35.48)	107 (65.64)	
MODS, *n* (%)				< 0.001
No	955 (98.56)	802 (99.50)	153 (93.87)	
Yes	14 (1.44)	4 (0.50)	10 (6.13)	
Stroke, *n* (%)				< 0.001
No	499 (51.50)	391 (48.51)	108 (66.26)	
Yes	470 (48.50)	415 (51.49)	55 (33.74)	
Cardiac arrest, *n* (%)				< 0.001
No	918 (94.74)	773 (95.91)	145 (88.96)	
Yes	51 (5.26)	33 (4.09)	18 (11.04)	
Complication, *n* (%)				
Coagulopathy, *n* (%)				< 0.001
No	922 (95.15)	784 (97.27)	138 (84.66)	
Yes	47 (4.85)	22 (2.73)	25 (15.34)	
DIC, *n* (%)				< 0.001
No	956 (98.66)	803 (99.63)	153 (93.87)	
Yes	13 (1.34)	3 (0.37)	10 (6.13)	
Sepsis, *n* (%)				< 0.001
No	830 (85.66)	760 (94.29)	70 (42.94)	
Yes	139 (14.34)	46 (5.71)	93 (57.06)	

### 3.3 Predictor selection using Boruta and LASSO for MDR-KP infection outcomes

**Figure 2:**
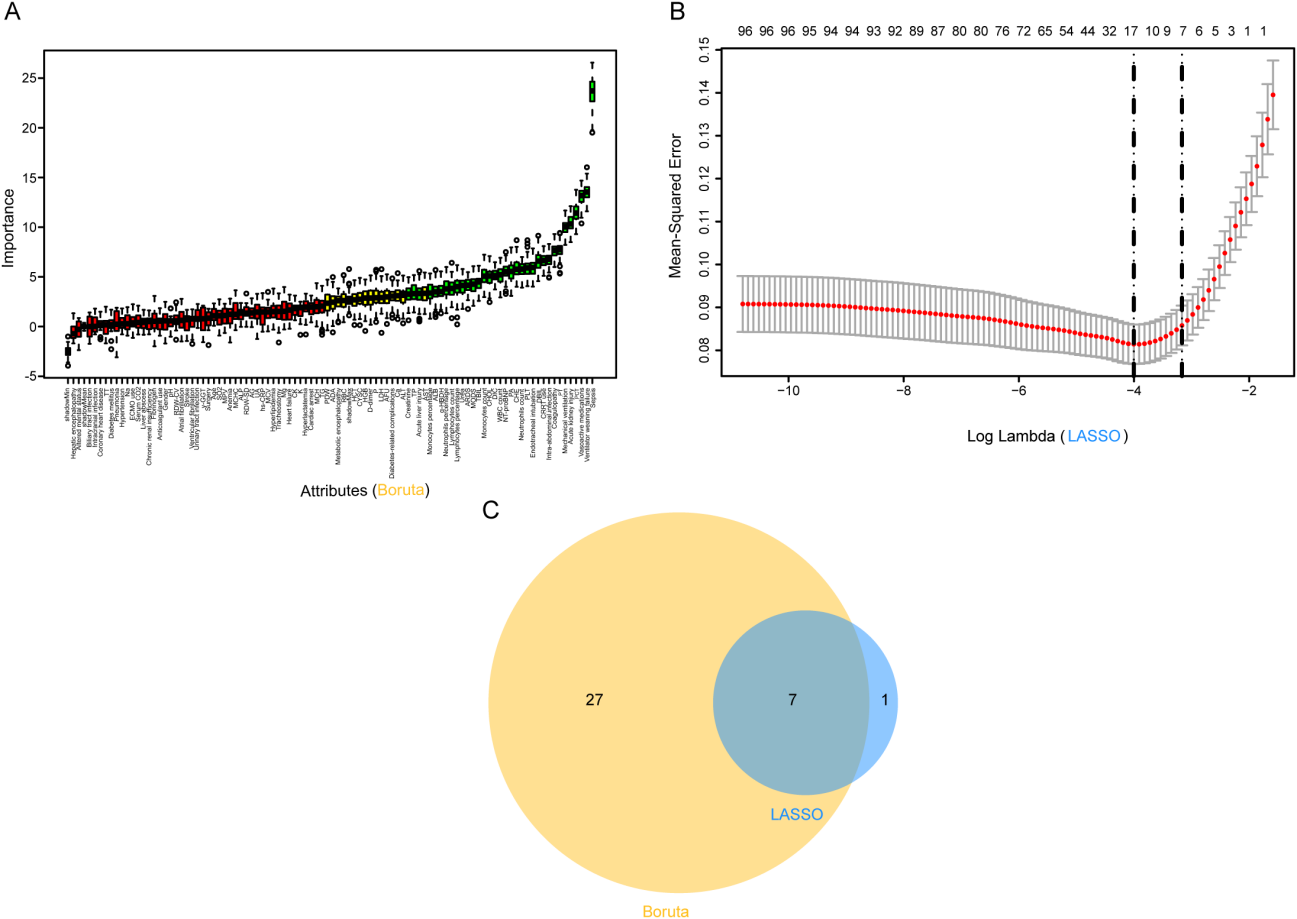
Predictor screening results. **(A)** Boruta; **(B)** Factor screening based on the LASSO regression model, with the left dashed line indicating the best lambda value for the evaluation metrics (lambda. min) and the right dashed line indicating the lambda value for the model where the evaluation metrics are in the range of the best value by one standard error (lambda.1se); **(C)** Boruta combined LASSO. LASSO, Least Absolute Shrinkage and Selection Operator.

Feature selection employed both Boruta algorithm and LASSO regression analysis. The Boruta algorithm identified 34 significant predictors, including laboratory parameters (cellular components, biochemical markers, and coagulation indices) and clinical factors (organ dysfunction indicators and interventions) (**Figure 2A**). LASSO regression yielded eight key predictive variables: mechanical ventilation, ventilator weaning failure, vasoactive medications, TG, intra-abdominal infection, acute kidney injury, PCT, and sepsis (**Figure 2B**). The intersection of these methodologies produced seven consistent predictors: mechanical ventilation, ventilator weaning failure, vasoactive medications, intra-abdominal infection, acute kidney injury, PCT, and sepsis. These variables were incorporated into the final predictive model (**Figure 2C**).

### 3.4 Comparison of machine learning algorithms for predicting MDR-KP-related septic shock

**Figure 3:**
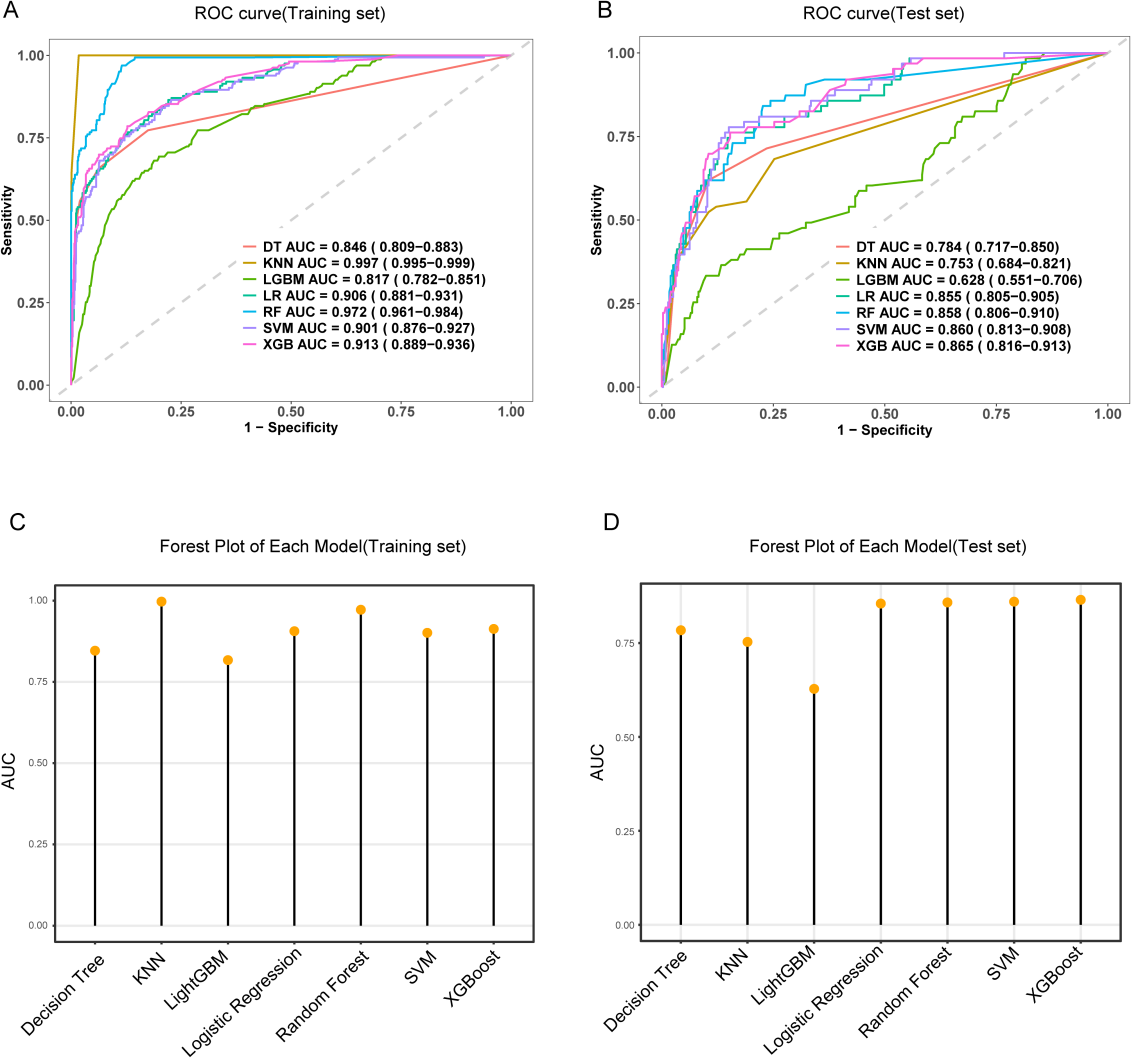
The performance and comparison of seven different predictive models. **(A)** The training set ROC curve; **(B)** The test set ROC curve; **(C)** Forest plot of AUC values in the training set; **(D)** Forest plot of AUC values in the test set.

Seven machine learning algorithms [Logistic Regression (LR), Decision Tree (DT), Random Forest (RF), XGBoost, SVM, KNN, and LightGBM] were implemented to predict MDR-KP-related septic shock using the seven key variables identified through combined LASSO and Boruta feature selection. Model optimization employed repeated 5-fold cross-validation with AUC-based parameter tuning. Performance evaluation incorporated multiple metrics: AUC, accuracy, sensitivity, specificity, PPV, NPV, and F1 score (**Figure 3**, **Table 2**). In the training cohort, KNN demonstrated superior performance (AUC: 0.997, accuracy: 0.986, sensitivity: 1.000, specificity: 0.983, PPV: 0.921, NPV: 1.000, F1: 0.959). However, in the test cohort, the Logistic Regression model was selected as optimal to minimize overfitting risk (AUC: 0.865, accuracy: 0.724, sensitivity: 0.810, specificity: 0.708, PPV: 0.331, NPV: 0.954, F1: 0.470).

**Table 2:** Predictive performance comparison of the seven types of machine learning algorithms.

Variables		Train Cohort					
	AUC	Accuracy	Sensitivity	Specificity	PPV	NPV	F1 score
Logistic Regression	0.906	0.794	0.865	0.779	0.442	0.966	0.585
Decision Tree	0.846	0.909	0.850	0.916	0.558	0.980	0.674
Random Forest	0.972	0.899	0.969	0.885	0.630	0.993	0.763
XGBoost	0.913	0.858	0.785	0.872	0.554	0.953	0.65
SVM	0.901	0.808	0.828	0.804	0.461	0.959	0.592
KNN	0.997	0.986	1.000	0.983	0.921	1.000	0.959
LightGBM	0.817	0.782	0.693	0.800	0.412	0.928	0.517

		Test Cohort					
Logistic Regression	0.865	0.724	0.810	0.708	0.331	0.954	0.470
Decision Tree	0.784	0.873	0.619	0.901	0.413	0.955	0.495
Random Forest	0.858	0.784	0.841	0.773	0.399	0.965	0.541
XGBoost	0.865	0.808	0.762	0.816	0.425	0.951	0.546
SVM	0.860	0.755	0.810	0.745	0.362	0.956	0.500
KNN	0.753	0.839	0.524	0.895	0.471	0.913	0.496
LightGBM	0.628	0.695	0.444	0.739	0.233	0.882	0.306

### 3.5 Development and validation of a logistic regression model for predicting MDR-KP-related septic shock

**Figure 4:**
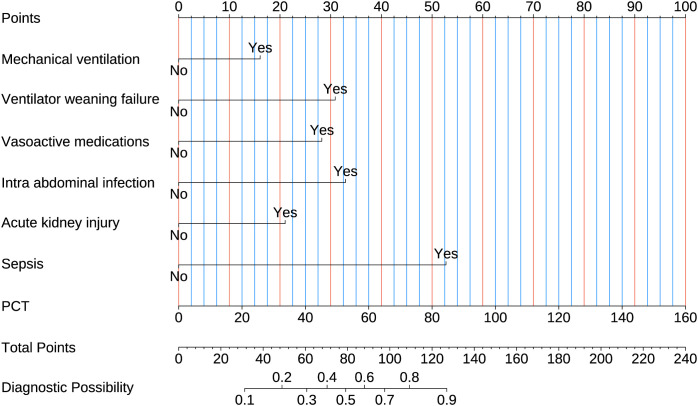
Nomogram used for predicting MDR-KP-induced septic shock. Logistic regression algorithm was used to establish nomogram. The final score is calculated as the sum of the individual scores of each of the ten variables included in the nomogram.

Multivariate and univariate logistic analyses identified seven significant predictors of MDR-KP-related septic shock: mechanical ventilation, ventilator weaning failure, intra-abdominal infection, acute kidney injury, vasoactive medications, PCT, and sepsis. The multivariate logistic regression coefficients and odds ratios are presented in **Table 3**. The predictive model was expressed as: Logit (P) = -3.634 + 0.029 (PCT) + 2.422 (Sepsis) + 0.965 (Acute kidney injury) + 1.512 (Intra-abdominal infection) + 1.297 (Vasoactive medications) + 1.418 (Ventilator weaning failure) + 0.738 (Mechanical ventilation). These variables were subsequently incorporated into a nomogram for visual prediction of MDR-KP-related septic shock probability (**Figure 4**).

**Table 3:** Univariate and Multivariate analysis of risk factors for MDR-KP-induced septic shock inpatients.

Characteristics	Univariate analysis				Multivariate analysis			
	Coef	OR	95% CI	*P* value	Coef	OR	95% CI	*P* value
Intercept					-3.634	0.026	0.016-0.041	<0.001
PCT	0.039	1.040	1.029-1.052	<0.001	0.029	1.029	1.016-1.042	<0.001
Sepsis	3.089	21.950	14.380-34.010	<0.001	2.422	11.269	6.890-18.630	<0.001
Acute kidney injury	1.871	6.496	4.508-9.392	<0.001	0.965	2.624	1.601-4.275	<0.001
Intra-abdominal infection	2.410	11.133	5.219-25.260	<0.001	1.512	4.534	1.638-13.210	0.004
Vasoactive medications	2.166	8.727	5.874-13.020	<0.001	1.297	3.660	2.135-6.275	<0.001
Ventilator weaning failure	2.821	16.796	8.681-34.650	<0.001	1.418	4.131	1.751-10.110	0.001
Mechanical ventilation	1.725	5.615	3.794-8.518	<0.001	0.738	2.093	1.246-3.552	0.006

CI, confidence interval; OR, odds ratio.

**Figure 5:**
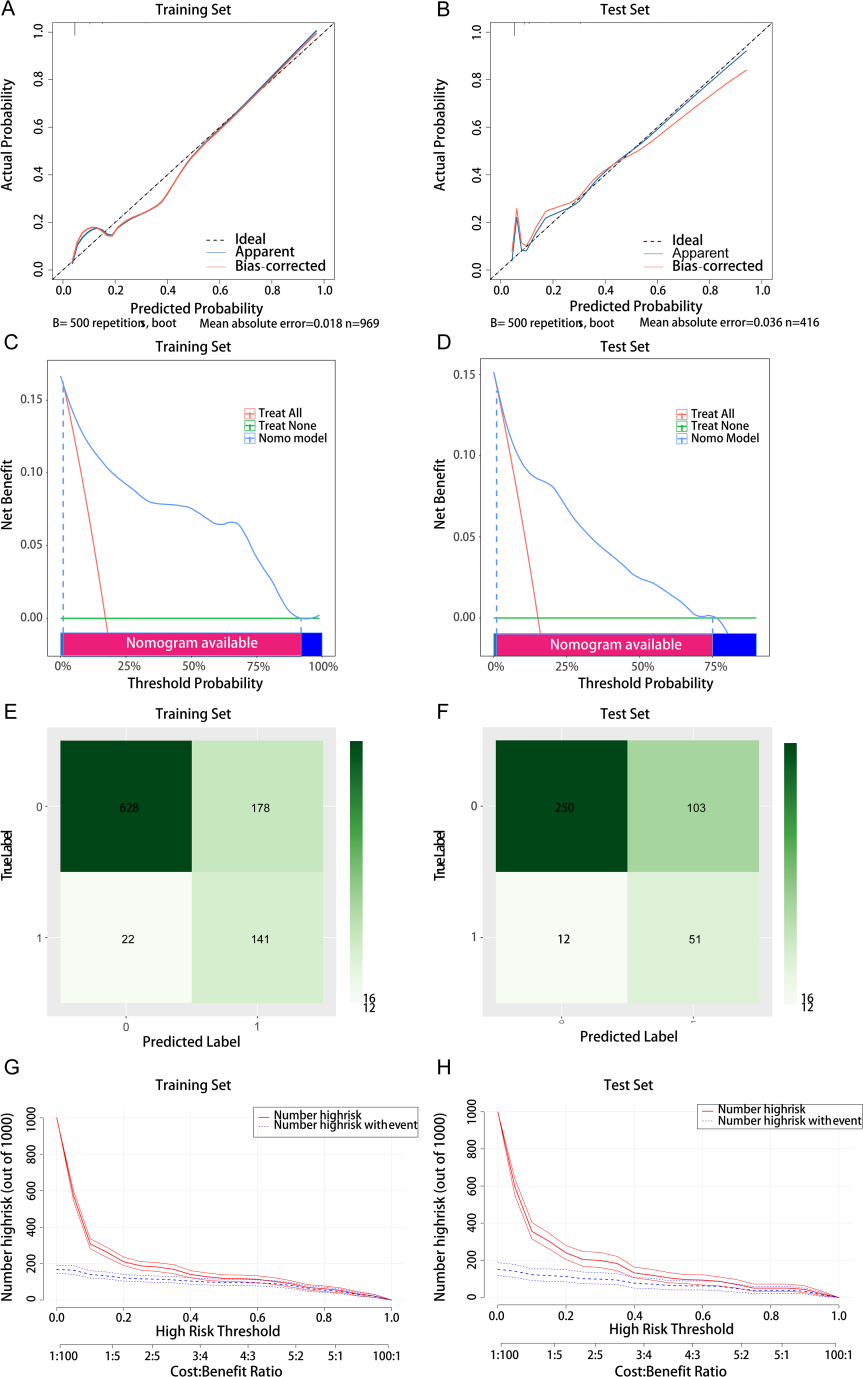
Comprehensive evaluation of the logistic regression model. **(A)** Calibration curve for the training set; **(B)** Calibration curve for the test set; **(C)** Decision curve analysis for the training set; **(D)** Decision curve analysis for the test set; **(E)** Confounding matrix for the training set; **(F)** Confounding matrix for the test set; **(G)** Clinical impact curve for the training set; **(H)** Clinical impact curve for the test set.

Comprehensive validation of the model’s predictive performance revealed robust capabilities across multiple assessment metrics. The model demonstrated exceptional discrimination in the training set with an AUC of 0.906 (**Figure 3A**), supported by strong calibration (Hosmer-Lemeshow test P = 0.065; **Figure 5A**). This performance was consistently maintained in the test set, achieving an AUC of 0.865 (**Figure 3B**) with adequate calibration (Hosmer-Lemeshow test P=0.069; **Figure 5B**). The model’s practical utility was confirmed through decision curve analysis, demonstrating superior net benefits across threshold probabilities of 0.01-0.92 in the training set (**Figure 5C**) and 0.01-0.75 in the test set (**Figure 5D**). Detailed confusion matrix analysis further validated the model’s performance, with the training set achieving 86.5% sensitivity and 77.9% specificity (628 true negatives, 141 true positives, 178 false positives, 22 false negatives; **Figure 5E**). Similar robust performance was observed in the test set, with 81.0% sensitivity and 70.8% specificity (250 true negatives, 51 true positives, 103 false positives, 12 false negatives; **Figure 5F**). Clinical impact curves (CIC) (**Figures 5G, H**) provided additional validation of the model’s clinical utility across varying cost-benefit ratios. Particularly noteworthy was the strong alignment between predicted high-risk cases and actual MDR-KP-related septic shock occurrences at threshold probabilities exceeding 40%, substantiating the model’s practical clinical value.

### 3.6 Nomogram with individual predictors for septic shock in MDR-KP infections

**Figure 6:**
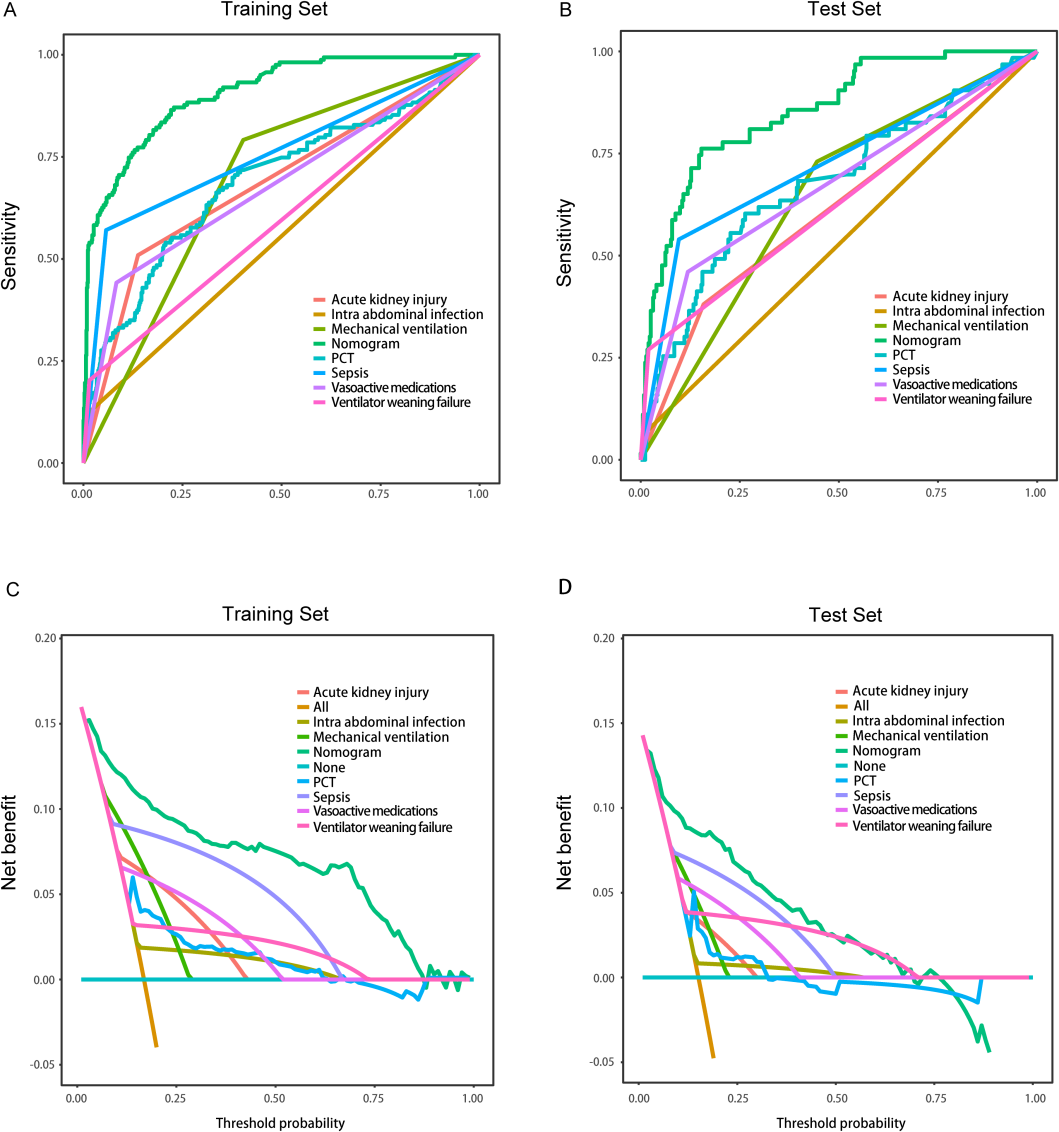
Comparison of the models in the entire study cohort. **(A)** Receiver operating characteristic curves of various models for the training set; **(B)** Receiver operating characteristic curves of various models the test set; **(C)** Decision curve analysis of various models for the training set; **(D)** Decision curve analysis of various models for the test set.

In the training set, the nomogram demonstrated superior discriminative ability (AUC = 0.906), and DeLong tests showed significant differences when compared with each individual predictor (all P < 0.001): intra-abdominal infection (AUC = 0.555), sepsis (AUC = 0.757), PCT (AUC = 0.690), vasoactive medications (AUC = 0.679), mechanical ventilation (AUC = 0.694), ventilator weaning failure (AUC = 0.594), and acute kidney injury (AUC = 0.686) (**Figure 6A**). Similarly, in the validation set, the nomogram achieved better predictive performance (AUC = 0.865), and DeLong tests confirmed significant differences compared to all individual predictors (all P < 0.001): intra-abdominal infection (AUC = 0.761), sepsis (AUC = 0.722), PCT (AUC = 0.676), vasoactive medications (AUC = 0.671), mechanical ventilation (AUC = 0.643), ventilator weaning failure (AUC = 0.625), and acute kidney injury (AUC = 0.611) (**Figure 6B**). Decision curve analyses confirmed the nomogram's enhanced clinical utility in both training (**Figure 6C**) and validation sets (**Figure 6D**), demonstrating consistently superior net benefit across varying threshold probabilities compared to single-factor models..

## 4 Discussion

This study successfully developed a robust machine learning model to predict the risk of septic shock in patients with multidrug-resistant Klebsiella pneumoniae (MDR-KP) infections. Employing a combined approach of LASSO regression and Boruta algorithm, we identified seven key predictors: PCT levels, sepsis, acute kidney injury, intra-abdominal infection, use of vasoactive medications, ventilator weaning failure, and mechanical ventilation. Logistic regression was the optimal method for model development, showing excellent performance in both training and validation phases.

Our predictive model aligns with and expands upon the findings of Cano et al. (56), who identified similar risk factors for MDR-KP infections but did not specifically explore outcomes related to septic shock. Conversely, our results provide a nuanced understanding of septic shock progression in these infections, diverging from Giannella et al. (57), who reported limited predictive utility of certain clinical parameters in similar contexts. This disparity may reflect differences in patient demographics, healthcare settings, and regional MDR-KP strains (58).

The clinical utility of our model is evidenced by its ability to facilitate early identification of high-risk patients, supporting timely and targeted interventions. This predictive capability allows for the stratification of patients into high-risk and low-risk categories based on their individual characteristics. Furthermore, incorporating patient stratification into clinical practice facilitates more precise and personalized care pathways. For example, high-risk patients identified by the model can receive personalized antimicrobial treatment plans tailored to their specific risk profiles, whereas low-risk patients can follow standardized treatment protocols, thereby minimizing unnecessary exposure to broad-spectrum antibiotics and reducing potential side effects. This approach aligns with the principles of precision medicine, which involves customizing treatment strategies according to each patient’s unique characteristics, thereby improving overall clinical outcomes and reducing healthcare costs. This approach is corroborated by Gutiérrez-Gutiérrez et al. (59), who highlighted the benefits of early intervention in managing MDR bacterial infections. Moreover, the model enhances antimicrobial stewardship by enabling precise risk stratification, which could potentially reduce unnecessary use of broad-spectrum antibiotics, aligning with recommendations by Gomez-Simmonds et al. (60). Our findings underscore the importance of specific monitoring parameters such as PCT and markers of acute kidney injury, which are crucial for early intervention and surveillance in MDR-KP-infected patients. These insights contribute significantly to the clinical management of these infections and are supported by robust model validation metrics including AUC, calibration, and decision curve analysis.

While the study underscores the value of the predictive model, the limitations should be acknowledged. One key limitation is the single-center and retrospective design, which may restrict the generalizability of the findings (61). Importantly, variations in MDR-KP strains and healthcare environments across different regions and institutions could influence the model’s applicability. Hence, we acknowledge that the applicability of the model to different populations and healthcare settings, especially those with distinct antimicrobial resistance patterns or patient demographics, may be limited by our current single-center design. To address this, we plan to conduct multi-center validation studies involving diverse cohorts and varying resistance profiles. Such efforts will allow us to confirm the model’s performance across heterogeneous environments, enhance its robustness, and improve its adaptability to a broader range of clinical scenarios. Furthermore, model performance might vary with regional differences in antimicrobial resistance patterns and treatment protocols, a limitation acknowledged in similar studies (62). Future research should focus on multi-center validation as recommended by Zarkotou et al. (63), to assess the model’s applicability in different healthcare settings. Prospective studies, following the methodological approach of Kontopoulou et al. (64), are also needed to confirm the model’s reliability and overcome retrospective biases. Integrating genomic data on MDR-KP strains could further refine the model’s accuracy and uncover strain-specific risk factors, while evaluating the model across diverse patient subgroups, as demonstrated in the stratification methodology by Papadimitriou-Olivgeris et al. (65), will ensure its effectiveness across varied clinical scenarios. Once high-risk patients are identified by the model, several key clinical interventions are recommended. These measures include strengthening infection control practices and promptly optimizing antimicrobial therapy. Prior to the finalization of microbiological results, it may be necessary to consult with infectious disease specialists to rapidly escalate or adjust empiric antibiotic regimens to swiftly control the source of infection. Early initiation of enhanced organ function monitoring, particularly for renal and circulatory support, should be guided by known risk factors such as procalcitonin (PCT) levels and acute kidney injury. Furthermore, high-risk patients may benefit from early admission to the intensive care unit (ICU), individualized mechanical ventilation strategies, careful evaluation of ventilator weaning readiness, and, if necessary, the early initiation of renal replacement therapy or vasoactive support to stabilize hemodynamics. Prompt imaging, surgical consultation, or drainage procedures for intra-abdominal infections can mitigate the progression to septic shock.

In light of the increasingly complex landscape of multidrug resistance, there is a growing need for predictive models capable of handling the multifaceted interplay among diverse resistance mechanisms, host factors, and evolving treatment strategies. Future models should consider incorporating a broader and more dynamic set of clinical and laboratory parameters, alongside advanced machine learning or deep learning frameworks, to capture non-linear and time-dependent patterns. Such enhanced models are expected to provide more accurate risk stratification and improve clinical decision-making, ultimately guiding precision therapy and patient management in the face of complex and rapidly changing MDR scenarios. Our predictive model not only deepens the understanding of MDR-KP-related septic shock but also offers a vital clinical tool for early risk assessment and management, potentially reducing mortality as noted by Hauck et al. (66). The methodologies developed here may also serve as a blueprint for constructing similar predictive models for other antimicrobial-resistant infections, advancing the field of precision medicine in infectious diseases.

## 5 Conclusion

This study presents a machine learning-based model that integrates a wide array of clinical and laboratory parameters to assess septic shock risk in patients infected with MDR-KP. By leveraging advanced predictive analytics, the model offers a precise tool for early risk identification, which is expected to improve clinical outcomes, optimize resource allocation, and support targeted treatment strategies. Future work will focus on validating the model across multiple centers with diverse patient populations and varying antimicrobial resistance patterns to ensure its generalizability and enhance its clinical impact. These methodologies not only enhance our current understanding but also pave the way for future innovations in managing antimicrobial-resistant infections.

## Data Availability

The original contributions presented in the study are included in the article/**Supplementary Material**. Further inquiries can be directed to the corresponding authors.
