# Supplementary material for: Developing and validating a machine learning model to predict multidrug-resistant Klebsiella pneumoniae-related septic shock
Source: Front Immunol. 2025 Jan 10;15:1539465. doi: 10.3389/fimmu.2024.1539465 (PMC11757138; doi:10.3389/fimmu.2024.1539465)
Supplement: Supplementary file 1 [file DataSheet1.docx]

**Supplementary materials**

**2. Materials and methods**

**2.6 Feature screening**

**2.6.1 Least absolute shrinkage and selection operator**

The Least Absolute Shrinkage and Selection Operator (LASSO) regression was employed for variable selection and model refinement. Implementation utilized the 'glmnet' package in R (family = "binomial", α = 1). Two lambda values were determined through cross-validation: lambda.min for minimal cross-validation error, and lambda.1se for model parsimony. Variables with non-zero coefficients were retained, addressing multicollinearity and overfitting challenges common in high-dimensional medical datasets.

**2.6.2 Boruta**

The Boruta algorithm (Version 8.0.0), a Random Forest-based feature selection method, was implemented to determine feature importance. The algorithm creates 'shadow attributes' for each original feature and iteratively compares their importance through 500 iterations or until reaching stability. Features consistently outperforming their shadow counterparts were identified as important. Results were extracted using 'attStats' function and processed through custom 'adjustdata' function, providing robust feature selection while minimizing false negatives.

**2.7 Machine learning algorithms**

**2.7.1 Logistic regression algorithm**

Logistic Regression (LR) with L2 regularization was employed to model the relationship between predictors and binary outcome. The model estimated outcome probability through logistic transformation of predictor combinations. Hyperparameters were optimized with regularization factor (C) = 1.0, maximum iterations = 100, and convergence tolerance = 0.0001 to balance performance and prevent overfitting.

**2.7.2 Decision tree**

Decision tree (DT) model using the CART (Classification and Regression Trees) algorithm was implemented to predict MDR-KP-associated septic shock risk. The model parameters were optimized with maximum tree depth = 5, minimum samples for node splitting = 20, and minimum leaf samples = 10, ensuring balance between model complexity and interpretability.

**2.7.3 Random forest algorithm**

Random Forest (RF) algorithm was implemented to identify non-linear associations and enhance model generalization. The model was configured with 20 trees using Gini index splitting criterion, unrestricted maximum depth, and zero minimum impurity reduction.

**2.7.4 Extreme gradient boosting**

Extreme Gradient Boosting (XGBoost) was implemented to predict MDR-KP-associated septic shock risk. The model was optimized with maximum tree depth = 6, learning rate = 0.1, 100 estimators, and L2 regularization (lambda = 1.0) to ensure robust pattern recognition while preventing overfitting.

**2.7.5 Support vector machine algorithm**

Support Vector Machine (SVM) with Radial Basis Function (RBF) kernel was employed for classification. The model was configured with regularization parameter C = 1.0 and tolerance = 0.001, optimizing decision boundary detection in high-dimensional space.

**2.7.6 K-Nearest neighbor algorithm**

K-Nearest Neighbors (KNN) algorithm was implemented with k = 5 and uniform weighting for sample classification. This non-parametric approach classifies based on majority voting of nearest neighbors, providing adaptive classification without explicit model training.

**2.7.7 Light gradient boosting machine**

Light Gradient Boosting Machine (LightGBM) was implemented for MDR-KP-associated septic shock risk prediction. The model was configured with maximum tree depth = 6, learning rate = 0.1, 100 boosting iterations, and L1 regularization (lambda = 0.5) to optimize pattern recognition while ensuring computational efficiency.
